# Elevated FGR protein expression identifies a high-risk subset of diffuse large B-cell lymphoma and a potential therapeutic target

**DOI:** 10.3389/fonc.2026.1688949

**Published:** 2026-04-16

**Authors:** Kexin Shen, Shiyu Jiang, Ailing Gui, Yichen Yan, Hui Sun, Jiachen Wang, Baohua Yu, Yizhen Liu, Qunling Zhang

**Affiliations:** 1 Department of Medical Oncology, Fudan University Shanghai Cancer Center, Shanghai, China; 2 Peking University Institute of Hematology, Peking University People’s Hospital, Beijing, China; 3 Department of Oncology, Shanghai Medical College, Fudan University, Shanghai, China; 4 Department of Cellular and Genetic Medicine, School of Basic Medical Sciences, Fudan University, Shanghai, China; 5 Department of Pathology, Fudan University Shanghai Cancer Center, Shanghai, China

**Keywords:** biomarker, diffuse large B-cell lymphoma, FGR, immunohistochemistry, prognosis, risk stratification, Src-family kinase

## Abstract

**Background:**

Diffuse large B-cell lymphoma (DLBCL) is the most common non-Hodgkin lymphoma, and despite effective first-line immunochemotherapy a substantial proportion of patients develop relapse/refractory (RR) disease, underscoring the need for practical biomarkers beyond clinical risk scores.

**Methods:**

We retrospectively evaluated FGR protein expression by immunohistochemistry (IHC) in 91 patients with *de novo* DLBCL treated with first-line R-CHOP. FGR staining was scored using an immunoreactive score (IRS), and high expression was defined as IRS ≥ 2. Associations with RR events and survival were assessed by Kaplan-Meier analysis and Cox regression, and predictive performance at 5 years was compared with the International Prognostic Index (IPI) using Receiver operating characteristic (ROC) analysis. External validation was performed using a publicly available gene-expression dataset.

**Results:**

High FGR expression was observed in 64 of 91 tumors (70.3%) and was associated with a higher 5-year RR rate (29.7% vs 7.4%, P = 0.021). In Kaplan-Meier analyses, progression-free survival (PFS) was 69.1% in the high-expression group versus 92.1% in the low-expression group (P = 0.093), and overall survival (OS) was 80.0% versus 96.2% (P = 0.050); separation was more pronounced in the non-germinal center B-cell (non-GCB) subgroup. In multivariable Cox regression, high FGR remained independently associated with inferior 5-year PFS (HR 4.73, 95% CI 1.03-21.65; P = 0.045) and showed a strong trend toward inferior 5-year OS (HR 6.52, 95% CI 0.79-53.99; P = 0.082). ROC analysis suggested that FGR and IPI had similar discrimination at 5 years in this cohort. In external validation, FGR mRNA expression was elevated in non-GCB cases, and high FGR expression (upper quartile) was associated with a trend toward inferior OS.

**Conclusion:**

Elevated FGR protein expression identifies a subset of DLBCL patients with increased treatment failure and inferior outcomes, particularly within non-GCB subtypes, supporting prospective validation in larger, less selected cohorts and further mechanistic studies of the FGR/Src-family kinase axis as a candidate therapeutic target.

## 1 Introduction

Diffuse large B-cell lymphoma (DLBCL) is the most common non-Hodgkin lymphoma, accounting for 30-40% of adult cases with an incidence of ~6–7 per 100,000 person-years (1, 2). Rituximab plus cyclophosphamide, doxorubicin, vincristine, and prednisone (R-CHOP) remains standard first-line immunochemotherapy and induces durable remission in 60-70% of patients (3). However, 20-40% experience relapse or refractory disease, and outcomes after early treatment failure remain poor, highlighting the need for improved risk stratification beyond conventional clinical scoring systems (4).

The International Prognostic Index (IPI) remains the most widely used clinical risk model, incorporating age, stage, lactate dehydrogenase (LDH), performance status, and extranodal involvement (5). Although still predictive, its discriminatory power is limited in the rituximab era, as patients with similar IPI scores often experience divergent outcomes (6, 7). Revised indices such as the NCCN-IPI and models integrating molecular factors modestly improve prediction (6), but substantial heterogeneity persists, highlighting an unmet need for biomarkers that reflect lymphoma biology (8–13).

Molecular subtyping based on cell-of-origin (COO) has been an important advance (14). Gene-expression profiling classifies DLBCL into germinal center B-cell-like (GCB) and activated B-cell-like (ABC) subtypes with distinct biology and outcomes (15, 16). Non-GCB cases typically respond poorly to R-CHOP and show inferior survival, often due to chronic active B-cell receptor (BCR) signaling and constitutive NF-κB activation driven by recurrent mutations such as MYD88 and CD79B (17). Immunohistochemistry-based algorithms, such as the Hans classifier, are widely used in clinical practice and provide prognostic information beyond the IPI (18). However, COO alone only partially explains outcome heterogeneity, and comprehensive genomic classifications, while informative, are difficult to implement routinely (19). Practical immunohistochemical biomarkers that capture aggressive disease biology are therefore needed (20).

Beyond tumor-intrinsic features, the tumor immune microenvironment is increasingly recognized as a determinant of DLBCL biology, treatment response, and prognosis. Immune checkpoint dysregulation has also been implicated in DLBCL; notably, PD-1 and PD-L1 expression patterns have been investigated in DLBCL patients and may correlate with clinicopathologic features and outcomes, underscoring the importance of tumor-immune interactions in disease progression (21).

SRC-family kinases (SFKs) are non-receptor tyrosine kinases that mediate signaling from receptors such as BCR and cytokine receptors to downstream survival pathways including NF-κB, PI3K/AKT, and MAPK (22). Aberrant SFK activity is implicated in tumor growth, survival, and drug resistance across hematologic malignancies (23). FGR (FGR proto-oncogene, Src family tyrosine kinase; also known as the Gardner-Rasheed feline sarcoma viral oncogene homolog) is normally expressed in myeloid-lineage cells but can be aberrantly induced in lymphoid contexts (24, 25). In acute myeloid leukemia (AML), FGR overexpression drives leukemic proliferation and resistance to therapy, while in lymphoma models it has been linked to resistance to histone deacetylase inhibitors (26, 27). These findings suggest that dysregulated FGR signaling may confer survival advantages and contribute to chemoresistance (28).

Despite evidence supporting a functional role of FGR in other hematologic malignancies, its prognostic significance in DLBCL remains unclear. Given the reliance of non-GCB type DLBCL on chronic active BCR signaling—which depends on downstream SFKs—and evidence that related kinases such as HCK are overexpressed in aggressive subtypes (29, 30), we hypothesized that elevated FGR expression may identify a higher-risk subset of DLBCL.

In this study, we examined FGR protein expression by immunohistochemistry in a uniformly treated cohort of newly diagnosed DLBCL patients receiving R-CHOP, and examined associations with clinicopathologic features and survival outcomes. We further evaluated whether FGR provides prognostic information beyond established indices such as IPI and COO, and performed external validation using a publicly available dataset. Together, this work investigates FGR as a practical biomarker linked to aggressive DLBCL biology and a potential therapeutic target for future study.

## 2 Methods

### 2.1 Study design and ethics

**Figure 1:**
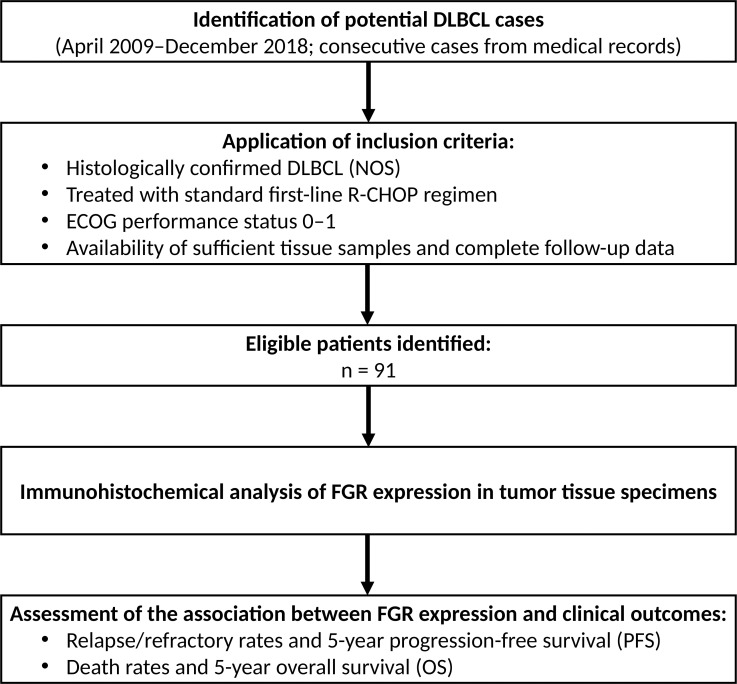
Flow diagram of patient inclusion and analysis. Flow chart showing cohort assembly and analysis steps. 91 patients were included for FGR IHC analysis and outcome evaluation. Patients were stratified by FGR expression, and clinical endpoints (PFS, OS) were assessed.

This study was a retrospective analysis of patients with newly diagnosed DLBCL treated at Fudan University Shanghai Cancer Center (Shanghai, China). We identified 91 consecutive cases diagnosed between April 2009 and December 2018 that met the inclusion criteria. The study workflow and patient selection are summarized in **Figure 1**. All patients received standard first-line R-CHOP immunochemotherapy. To minimize confounding by severe functional impairment and to ensure treatment comparability, only patients with an ECOG performance status of 0–1 at diagnosis were included. Patients with primary mediastinal B-cell lymphoma or transformed indolent lymphoma were excluded, focusing on *de novo* “DLBCL, not otherwise specified” cases per the World Health Organization (WHO) classification (2008 edition). Due to the retrospective design and the era of diagnosis, formal fluorescence *in situ* hybridization (FISH) testing for MYC, BCL2, or BCL6 gene rearrangements was not routinely performed on the archival samples; therefore, a small number of high-grade B-cell lymphomas with MYC and BCL2 and/or BCL6 rearrangements (“double-hit”/”triple-hit”) could have been inadvertently included. In addition, MYC/BCL2 co-expression by immunohistochemistry (double-expressor status) was not routinely assessed in the pretreatment period and thus could not be incorporated into the current analyses. The study was conducted in accordance with the Declaration of Helsinki and was approved by the Ethics Committee of Fudan University Shanghai Cancer Center (Institutional Review Board of Fudan University Shanghai Cancer Center, Approval No. 050432-4-1212B, Shanghai, China). Written informed consent for use of tumor samples and clinical data was obtained from all patients.

### 2.2 Patient cohort and clinical data

All 91 cases were pathologically confirmed as DLBCL by expert hematopathologists at the time of diagnosis based on morphological and immunophenotypic criteria. Clinical data were extracted from electronic medical records, including demographics (age, sex), Ann Arbor stage, presence of B symptoms, bulky disease status (tumor mass ≥10 cm), serum lactate dehydrogenase (LDH) level, extranodal involvement, bone marrow involvement, and IPI score (calculated as the sum of risk factors present). Each patient’s COO subtype (GCB vs non-GCB) was determined by immunohistochemistry as described below. Treatment responses were assessed from imaging and clinical evaluations after therapy. Response was defined according to standard International Working Group criteria, with complete response (CR) requiring resolution of disease on PET/CT and partial response (PR) indicating >50% tumor reduction. Refractory disease was defined as failure to achieve CR/PR with first-line therapy, and relapse was defined as disease progression after an initial CR. Progression-free survival (PFS) was calculated from diagnosis to the time of progression, relapse, or death from any cause. Overall survival (OS) was calculated from diagnosis to death from any cause. Follow-up was updated through December 31, 2020, and the median follow-up among survivors was 83.3 months (range, 2.7-117.6 months).

### 2.3 Histopathology and immunohistochemistry

Formalin-fixed, paraffin-embedded (FFPE) tumor tissue from diagnostic biopsies was used for all immunohistochemical analyses. Hematoxylin and eosin (H&E) slides were reviewed to confirm the diagnosis and assess tumor content. For immunohistochemistry, 4-μm thick FFPE tissue sections were deparaffinized and rehydrated, followed by heat-induced epitope retrieval (citrate buffer, pH 6.0, at 95 °C for 20 minutes). Endogenous peroxidase activity was blocked with 3% hydrogen peroxide. Sections were incubated with an anti-FGR monoclonal antibody (Cell Signaling Technology, Cat#2755; 1:100) for 60 minutes at room temperature. An avidin-biotin complex detection system (VECTASTAIN Elite ABC kit, Vector Laboratories) with diaminobenzidine (DAB) chromogen was used to visualize antibody binding. Slides were counterstained with hematoxylin. Appropriate positive and negative controls were included in each run.

Two board-certified hematopathologists (B.Y. and Q.Z.) independently evaluated the FGR staining under blinded conditions. FGR expression was semi-quantitatively scored using an immunoreactive score (IRS) incorporating both staining intensity and the percentage of positive tumor cells (31). Staining intensity in tumor cells was scored as 0 (negative), 1 (weak), 2 (moderate), or 3 (strong). The percentage of positive tumor cells was scored as follows: 0 = 0%, 1 = 1-24%, 2 = 25-49%, 3 = 50-74%, and 4 = ≥75%. The IRS was calculated as intensity score × percentage score (range, 0-12). Based on initial review and supported by Receiver operating characteristic (ROC)/Youden exploration across plausible IRS thresholds for 5-year death and relapse/refractory (RR) endpoints (**Supplementary Table 1**), IRS ≥2 was used to define high FGR expression. The two pathologists’ scores were highly concordant. In cases of discrepancy, slides were re-reviewed using a dual-headed microscope to reach a consensus score for analysis.

In addition to FGR, immunohistochemical staining for CD10, BCL6, and MUM1 was performed as part of the diagnostic workup to determine COO subtype. The Hans algorithm was applied: cases positive for CD10 (≥30% of tumor cells) were classified as GCB subtype; cases that were CD10-negative but BCL6-positive and MUM1-negative were also classified as GCB; all other cases including those MUM1-positive and/or CD10 and BCL6 double-negative were classified as non-GCB subtype.

### 2.4 Outcome definitions and statistical analysis

Clinical outcomes were defined as follows. PFS was the time from diagnosis to the first occurrence of disease progression, relapse, or death from any cause. OS was the time from diagnosis to death from any cause. Patients without an event were censored at the date of last follow-up. RR rate refers to the cumulative incidence of either primary refractory disease or relapse after an initial remission; fixed-time RR endpoints were evaluated at 5 years from diagnosis.

Kaplan-Meier curves were generated for PFS and OS and compared using the log-rank test. Cox proportional hazards regression was used for univariable and multivariable analyses, reporting hazard ratios (HR) with 95% confidence intervals (CI). Given the limited number of deaths, multivariable models were kept parsimonious and interpreted cautiously with emphasis on effect direction and uncertainty. For discrimination analyses, ROC curves were constructed for binary 5-year endpoints (5-year RR and 5-year death), and AUCs with 95% CIs were reported; AUCs were compared using DeLong’s test where applicable (32). All statistical analyses were performed using SPSS version 26.0 (IBM) and R software (version 4.2.2) with packages for survival and ROC analyses. A two-tailed P < 0.05 was considered statistically significant.

### 2.5 External validation in a public DLBCL dataset

To assess the external generalizability of our findings, we analyzed the publicly available DLBCL cohort GSE31312. FGR mRNA expression was represented by probe 208438_s_at (Affymetrix HG-U133 Plus 2.0). Associations between FGR expression and COO were tested using the Wilcoxon rank-sum test and logistic regression. Prognostic associations with OS and PFS were evaluated using Cox proportional hazards models with FGR modeled continuously (z-scored) and dichotomized at the cohort upper quartile.

## 3 Results

### 3.1 Patient characteristics

Ninety-one patients with newly diagnosed DLBCL met the inclusion criteria. Baseline clinical and pathological characteristics are summarized in **Table 1**. The mean age at diagnosis was 48 years (range 21-73), and 18 patients (19.8%) were older than 60 years. Most patients had an ECOG performance status of 0 (64.8%), and the remainder had ECOG 1 (35.2%). Fifty-nine patients (64.8%) presented with stage I-II disease and 32 (35.2%) with stage III-IV disease. Extranodal involvement was observed in 47 patients (51.6%). Elevated serum LDH (>250 U/L) was present in 25 patients (27.5%), and bulky disease (tumor mass ≥10 cm) was noted in 21 patients (23.1%). IPI scores were skewed toward lower risk: 64 patients (70.3%) had IPI 0-1, 26 (28.6%) had IPI 2-3, and 1 (1.1%) had IPI 4. By COO classification using the Hans IHC algorithm, 37 cases (40.7%) were GCB subtype and 54 (59.3%) were non-GCB subtype.

**Table 1:** Baseline characteristics of DLBCL patients stratified by FGR expression.

Variables	Total (n = 91)	Low FGR expression (n = 27)	High FGR expression (n = 64)	p
Gender, n (%)				0.568
Male	48 (52.7)	13 (48.1)	35 (54.7)	
Female	43 (47.3)	14 (51.9)	29 (45.3)	
Age, Mean ± SD	48.2 ± 12.8	49.3 ± 11.6	47.7 ± 13.4	0.606
Age, n (%)				0.440
> 60	73 (80.2)	23 (85.2)	50 (78.1)	
60	18 (19.8)	4 (14.8)	14 (21.9)	
ECOG, n (%)				0.812
= 0	59 (64.8)	18 (66.7)	41 (64.1)	
= 1	32 (35.2)	9 (33.3)	23 (35.9)	
Stage, n (%)				0.232
= I	27 (29.7)	11 (40.7)	16 (25)	
= II	32 (35.2)	10 (37)	22 (34.4)	
= III	15 (16.5)	4 (14.8)	11 (17.2)	
= IV	17 (18.7)	2 (7.4)	15 (23.4)	
Stage, n (%)				0.093
I~II	59 (64.8)	21 (77.8)	38 (59.4)	
III~IV	32 (35.2)	6 (22.2)	26 (40.6)	
LDH, Median (IQR)	183.0 (143.5, 271.0)	179.0 (141.0, 274.5)	186.5 (145.5, 262.2)	0.831
LDH, n (%)				0.765
> 250	66 (72.5)	19 (70.4)	47 (73.4)	
250	25 (27.5)	8 (29.6)	17 (26.6)	
Extranodal, n (%)				0.020
No	44 (48.4)	8 (29.6)	36 (56.2)	
Yes	47 (51.6)	19 (70.4)	28 (43.8)	
IPI, n (%)				0.135
= 0	46 (50.5)	14 (51.9)	32 (50)	
= 1	18 (19.8)	8 (29.6)	10 (15.6)	
= 2	17 (18.7)	3 (11.1)	14 (21.9)	
= 3	9 (9.9)	1 (3.7)	8 (12.5)	
= 4	1 (1.1)	1 (3.7)	0 (0)	
Bulky Disease, n (%)				0.335
No	70 (76.9)	19 (70.4)	51 (79.7)	
Yes	21 (23.1)	8 (29.6)	13 (20.3)	
Cell of Origin, n (%)				0.063
GCB	37 (40.7)	7 (25.9)	30 (46.9)	
Non-GCB	54 (59.3)	20 (74.1)	34 (53.1)	
Relapse/Refractory, n (%)				0.136
No	68 (74.7)	23 (85.2)	45 (70.3)	
Yes	23 (25.3)	4 (14.8)	19 (29.7)	
5-year Relapse/Refractory, n (%)				0.021
No	70 (76.9)	25 (92.6)	45 (70.3)	
Yes	21 (23.1)	2 (7.4)	19 (29.7)	
Death, n (%)				0.057
No	77 (84.6)	26 (96.3)	51 (79.7)	
Yes	14 (15.4)	1 (3.7)	13 (20.3)	
5-year Death, n (%)				0.098
No	78 (85.7)	26 (96.3)	52 (81.2)	
Yes	13 (14.3)	1 (3.7)	12 (18.8)	
PFS (days), Mean ± SD	1965.4 ± 1081.6	2345.7 ± 868.5	1804.9 ± 1127.7	0.029
5-year PFS (days), Mean ± SD	1410.8 ± 654.5	1653.1 ± 481.4	1308.6 ± 693.2	0.021
OS (days), Mean ± SD	2122.6 ± 958.9	2398.1 ± 850.9	2006.5 ± 984.2	0.075
5-year OS (days) Mean ± SD	1524.4 ± 544.6	1665.0 ± 469.3	1465.0 ± 566.4	0.110

Clinical and pathological features of 91 patients, overall and by FGR-high vs. FGR-low subgroups. Variables include age, sex, stage (I/II vs. III/IV), extranodal involvement, LDH, IPI score, bulky disease, and COO subtype.

When stratified by FGR expression status (IRS ≥2 vs <2), baseline differences were similar between groups (**Table 1**). Extranodal involvement was more frequent in the FGR-low group than the FGR-high group (70.4% vs 43.8%, P = 0.020). There was also a trend toward different COO distribution between groups (P = 0.063), while other variables including age, stage, ECOG, LDH, IPI, and bulky disease were not significantly different.

### 3.2 FGR expression by immunohistochemistry

**Figure 2:**
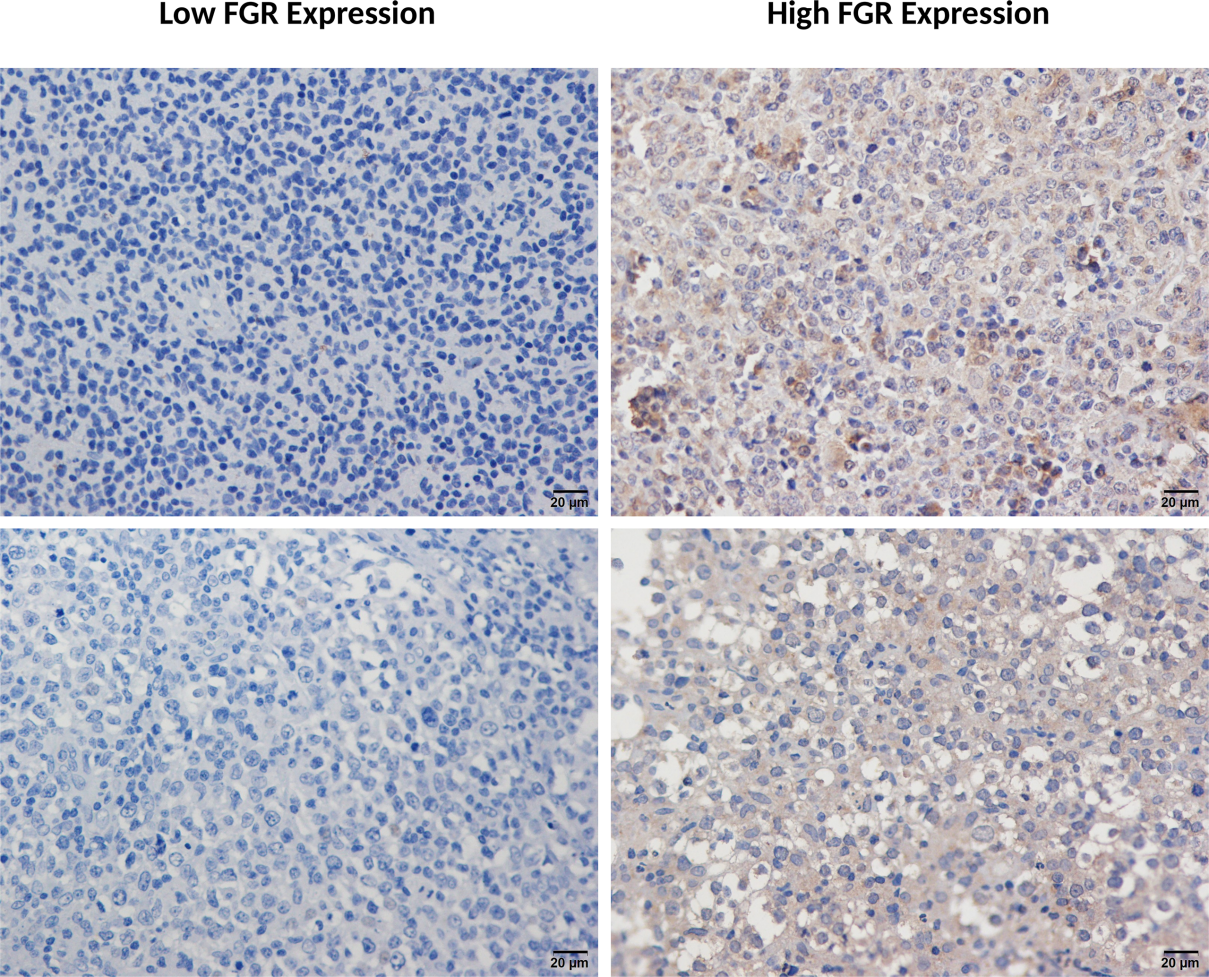
Immunohistochemical detection of FGR in DLBCL tumor samples. Representative immunohistochemical staining of FGR in DLBCL tissues (400×). Left panels show low FGR expression (IRS <2), characterized by weak and/or focal cytoplasmic staining in lymphoma cells, whereas right panels show high FGR expression (IRS ≥2), characterized by more diffuse and stronger cytoplasmic staining. Two representative fields are shown per group. Scale bars, 20 μm.

Using predefined IHC scoring criteria, 64 of 91 patients (70.3%) were classified as FGR-high (IRS ≥2), while 27 (29.7%) were FGR-low (IRS <2). ROC/Youden exploration across plausible IRS thresholds supported IRS = 2.0 as the optimal cutpoint for both 5-year death and 5-year RR, consistent with defining FGR-high as IRS ≥2 (**Supplementary Table 1**). Representative IHC images are shown in **Figure 2**. Staining was predominantly cytoplasmic with occasional membranous accentuation, consistent with FGR being a SFK localized to the inner plasma membrane. Background benign cells such as histiocytes and granulocytes also stained for FGR, reflecting normal myeloid expression, but were morphologically distinct from neoplastic B cells and were excluded from scoring.

We next assessed the relationship between FGR expression and COO subtype. FGR-high expression was observed in 30 of 37 (81.1%) GCB cases and 34 of 54 (63.0%) non-GCB cases, although the difference did not reach statistical significance (P = 0.063). Notably, very high IRS values (e.g., IRS ≥ 6) were enriched among non-GCB cases, suggesting that a subset of non-GCB tumors may exhibit particularly strong FGR upregulation.

External validation was performed in the public dataset GSE31312. In this cohort, FGR mRNA expression (probe 208438_s_at) was significantly higher in non-GCB than GCB DLBCL (Wilcoxon P = 5.15×10^−5^). In logistic regression, each 1-SD increase in FGR expression was associated with the non-GCB subtype (OR = 1.43, 95% CI 1.18-1.75; P = 3.94×10^−4^). In survival analyses, patients in the upper quartile of FGR expression showed a trend toward inferior OS compared with the remainder of the cohort (HR = 1.34, 95% CI 0.96-1.87; P = 0.088), while no association was observed for PFS (HR = 1.12, 95% CI 0.80-1.57; P = 0.510) (**Supplementary Figure 1**).

### 3.3 Clinical outcomes and association with FGR

During follow-up, 23 patients (25.3%) developed primary refractory disease or relapse, and 14 patients (15.4%) died. At 5 years, 21 patients (23.1%) had experienced RR events and 13 patients (14.3%) had died (**Table 1**).

**Figure 3:**
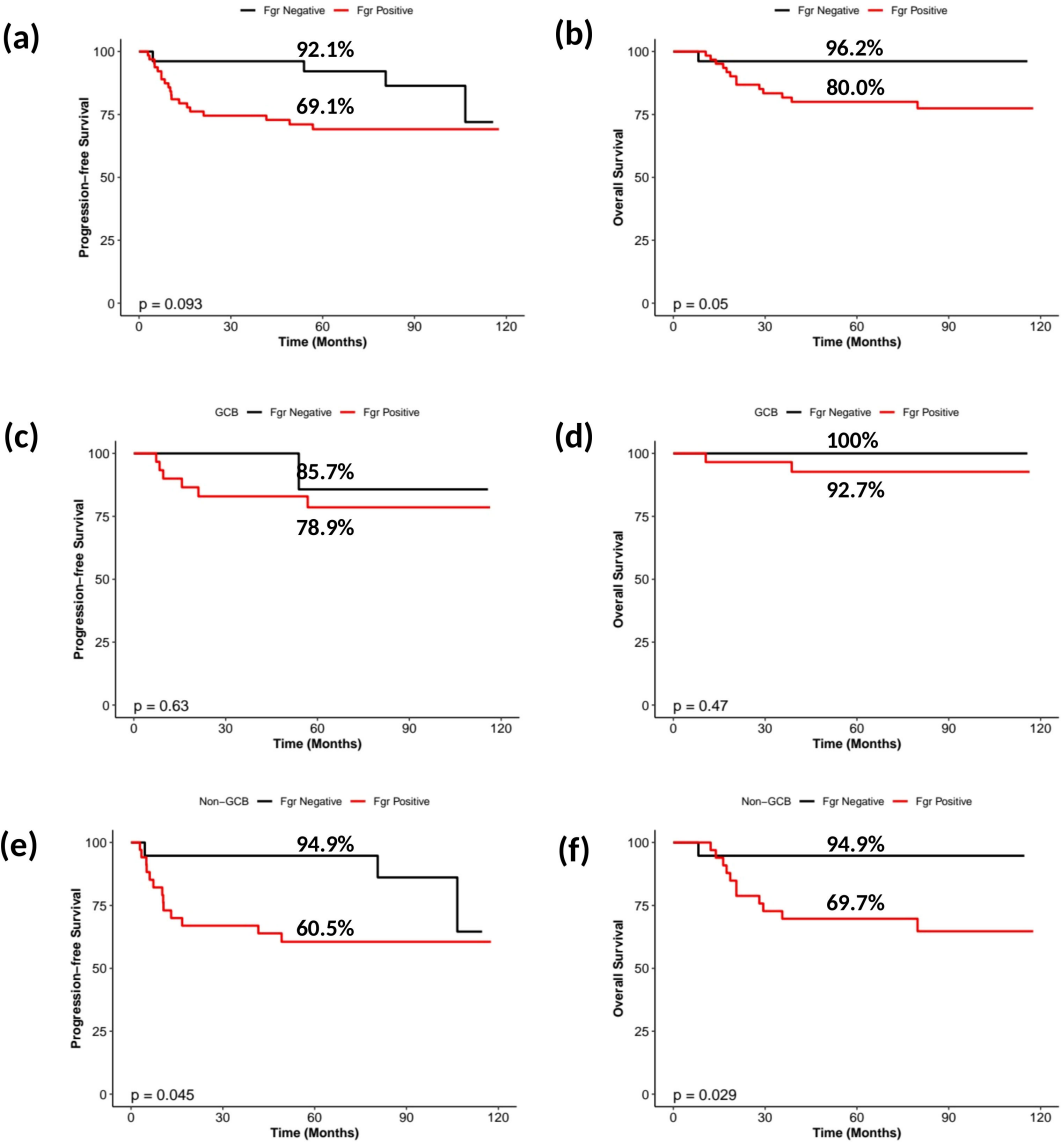
Kaplan-Meier survival curves stratified by FGR expression. Kaplan-Meier survival analyses according to FGR expression. **(A)** PFS in the entire cohort; **(B)** OS in the entire cohort; **(C)** PFS in GCB subtype; **(D)** OS in GCB subtype; **(E)** PFS in non-GCB subtype; **(F)** OS in non-GCB subtype.

We next evaluated outcomes by FGR expression status. Patients with FGR-high tumors experienced significantly higher treatment failure rates than those with FGR-low tumors. Specifically, the 5-year RR rate was 29.7% in the FGR-high group compared with 7.4% in the FGR-low group (P = 0.021). Consistently, mean PFS was shorter in the FGR-high group (mean 1805 vs 2346 days, P = 0.029; **Table 1**). Kaplan-Meier analysis showed inferior PFS and borderline inferior OS in FGR-high patients (5-year PFS 69.1% vs 92.1%, log-rank P = 0.093; 5-year OS 80.0% vs 96.2%, P = 0.050) (**Figures 3A, B**). OS showed a similar direction in mean survival time but did not reach conventional statistical significance in this dataset, likely reflecting the limited number of deaths.

To evaluate FGR in the context of other prognostic variables, we performed Cox regression analyses for 5-year PFS and OS (**Table 2**, **3**). On univariate analysis, high FGR expression was associated with inferior 5-year PFS (HR 4.57, 95% CI 1.06-19.64, P = 0.041) and remained independently associated with 5-year PFS in multivariate analysis (HR 4.73, 95% CI 1.03-21.65, P = 0.045). For 5-year OS, high FGR showed a trend toward worse outcomes (univariate HR 5.45, 95% CI 0.71-41.9, P = 0.103; multivariate HR 6.52, 95% CI 0.79-53.99, P = 0.082). Elevated LDH (>250 U/L), ECOG 1, and stage IV disease were adverse predictors of 5-year OS in univariate analysis.

**Table 2:** Univariate and multivariate Cox regression analysis of prognostic factors for 5-year PFS.

Univariate analysis			Multivariate analysis		
Variables	HR (95%CI)	p	Variables	HR (95%CI)	p
Age > 60 vs ≤ 60	1.7 (0.66,4.39)	0.271	Age ≤ 60	1(Ref)	
			Age > 60	1.03 (0.35,3.04)	0.956
Stage I	1(Ref)		Stage I	1(Ref)	
Stage II	1.79 (0.45,7.15)	0.412	Stage II	1.34 (0.32,5.61)	0.692
Stage III	2.73 (0.61,12.21)	0.19	Stage III	1.83 (0.36,9.34)	0.469
Stage IV	5.62 (1.49,21.25)	0.011	Stage IV	2.78 (0.61,12.62)	0.186
			Trend test	1.42 (0.89,2.25)	0.138
ECOG 1 vs 0	2.8 (1.18,6.65)	0.02	ECOG0	1(Ref)	
			ECOG1	2.13 (0.84,5.38)	0.111
LDH > 250 vs ≤ 250	1.9 (0.79,4.59)	0.154	LDH ≤ 250	1(Ref)	
			LDH > 250	1.26 (0.45,3.56)	0.663
non-GCB vs GCB	1.53 (0.62,3.8)	0.355	GCB	1(Ref)	
			non-GCB	1.7 (0.65,4.45)	0.282
FGR high vs low expression	4.57 (1.06,19.64)	0.041	FGR low expression	1(Ref)	
			FGR high expression	4.73 (1.03,21.65)	0.045

HR and 95% CI are shown for age, stage, ECOG performance status, serum LDH, COO subtype, and FGR expression.

**Table 3:** Univariate and multivariate Cox regression analysis of prognostic factors for 5-year OS.

Univariate analysis			Multivariate analysis		
Variables	HR (95%CI)	p	Variables	HR (95%CI)	p
Age > 60 vs ≤ 60	2.81 (0.92,8.6)	0.07	Age ≤ 60	1(Ref)	
			Age > 60	1.49 (0.41,5.44)	0.546
Stage I	1(Ref)		Stage I	1(Ref)	
Stage II	4.4 (0.51,37.65)	0.176	Stage II	2.16 (0.22,20.94)	0.505
Stage III	3.94 (0.36,43.51)	0.263	Stage III	1.22 (0.08,17.5)	0.886
Stage IV	8.9 (1.04,76.25)	0.046	Stage IV	1.74 (0.14,21.46)	0.664
			Trend test	1.03 (0.53,1.97)	0.939
ECOG 1 vs 0	3.08 (1.01,9.41)	0.049	ECOG0	1(Ref)	
			ECOG1	2.27 (0.63,8.24)	0.212
LDH > 250 vs ≤ 250	4.88 (1.6,14.95)	0.005	LDH ≤ 250	1(Ref)	
			LDH > 250	3.66 (0.91,14.65)	0.067
non-GCB vs GCB	3.95 (0.88,17.85)	0.074	GCB	1(Ref)	
			non-GCB	2.82 (0.56,14.13)	0.208
FGR high vs low expression	5.45 (0.71,41.9)	0.103	FGR low expression	1(Ref)	
			FGR high expression	6.52 (0.79,53.99)	0.082

HR and 95% CI are shown for age, stage, ECOG performance status, serum LDH, COO subtype, and FGR expression.

In exploratory subgroup analyses, the association between high FGR and inferior outcomes appeared more pronounced in the non-GCB subgroup. Among non-GCB patients, 5-year PFS was 60.5% in the FGR-high subgroup versus 94.9% in the FGR-low subgroup (P = 0.045), and 5-year OS was 69.7% versus 94.9% (P = 0.029) (**Figures 3E, F**). In contrast, within the GCB subtype, differences were not statistically significant (**Figures 3C, D**). These results suggest that high FGR may identify a particularly high-risk subset within the already aggressive subtype. Given the limited number of events, these subgroup findings should be interpreted as hypothesis-generating and warrant confirmation in larger cohorts.

### 3.4 Comparison of FGR with IPI

**Figure 4:**
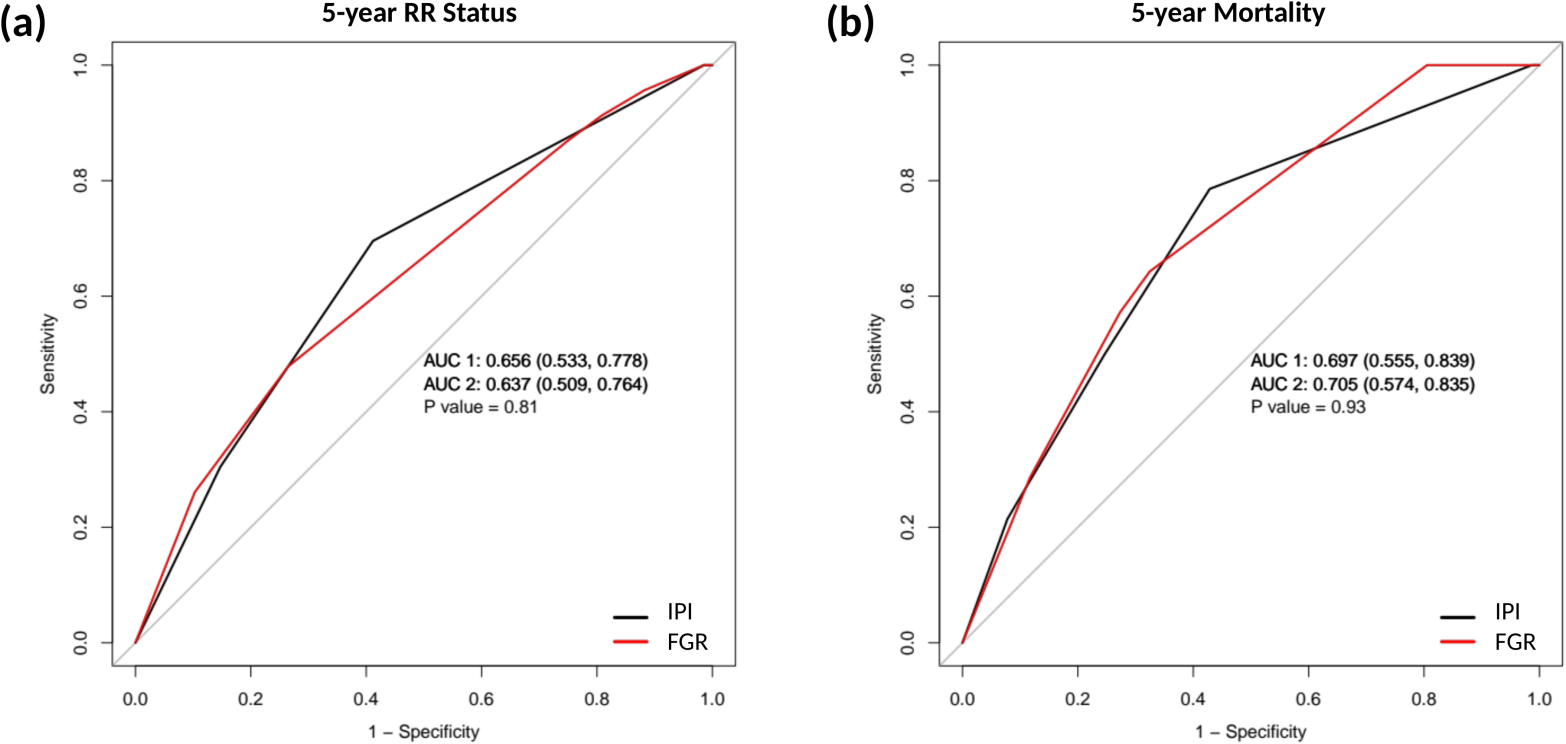
ROC analysis comparing prognostic discrimination at 5 years.ROC curves comparing IPI and FGR expression for 5-year endpoints. **(A)** Predicting 5-year relapse/refractory (RR) status; **(B)** predicting 5-year mortality (death).

Given that IPI is the standard clinical prognostic index, we compared the predictive performance of FGR expression versus the IPI using ROC analyses at 5 years (**Figure 4**). For predicting 5-year RR status, the AUC for FGR was 0.637 (95% CI: 0.51-0.76), which was very similar to the AUC for IPI at 0.656 (95% CI: 0.53-0.78). For 5-year overall mortality, FGR’s AUC was 0.705 (0.57-0.83) versus IPI’s AUC 0.697 (0.56-0.84). Statistically, there was no significant difference between the prognostic accuracy of FGR and IPI for these endpoints. In other words, as a single variable, tumor FGR expression had predictive power on par with the multi-factor IPI in this cohort. This finding is remarkable considering the IPI incorporates five clinical parameters whereas FGR is one biomarker; it highlights FGR’s potential value as a risk indicator.

## 4 Discussion

### 4.1 Key findings and clinical relevance

In this study, we report clinical evidence that elevated tumor-cell FGR protein expression is associated with increased treatment failure and inferior outcomes in DLBCL. High FGR expression was common (70.3%) and was associated with a substantially higher 5-year RR rate (29.7% vs 7.4%). In Cox regression, high FGR remained independently associated with inferior PFS and showed a consistent (but not statistically significant) trend toward inferior OS, with wide confidence intervals reflecting the limited number of deaths. Collectively, these findings suggest that FGR expression may capture an aspect of aggressive biology not fully reflected by conventional clinical variables.

Notably, the adverse impact of FGR was most pronounced in the non-GCB subtype of DLBCL. Within the non-GCB subset, FGR-high cases had dismal outcomes (only ~70% 5-year OS) despite R-CHOP, whereas non-GCB cases with low FGR had outcomes approaching those of GCB patients. This indicates that FGR expression helps stratify risk even within the clinically heterogeneous non-GCB category. Our findings add to the growing body of literature seeking to refine DLBCL prognostication by incorporating tumor-intrinsic biomarkers (14). While markers like double-hit status (MYC/BCL2 rearrangements) and co-expression of BCL2/MYC proteins have been shown to predict poor outcome, these primarily apply to a minority of GCB cases (33, 34). By contrast, FGR appears to identify high-risk biology particularly in the non-GCB subtypes, which is an area of unmet need since non-GCB/ABC-enriched DLBCL generally has worse outcomes and fewer reliable biomarkers (35).

### 4.2 Biological implications and mechanistic hypotheses

The preferential upregulation of FGR in the non-GCB/ABC subtype likely reflects underlying oncogenic pathways active in this subtype. ABC-DLBCL is characterized by chronic active BCR signaling and constitutive NF-κB activation, often driven by mutations in MYD88, CD79B, CARD11, and other components of the BCR/TLR signaling axis (23, 36). These signaling pathways can induce transcriptional programs that include upregulation of certain tyrosine kinases. Prior genomic studies have noted that ABC-DLBCL frequently harbors copy number gains on chromosome 19p13, a region that includes the gene SPIB, an ETS-family transcription factor essential for ABC cell survival (37). SPIB is markedly more expressed in ABC- than GCB-DLBCL and helps drive the plasmablastic transcriptional state of ABC cells. It has been reported that SPIB (and a related ETS factor, EHF) can regulate genes involved in endocytosis and vesicle trafficking, potentially including members of the Src-kinase family such as HCK (23). Although FGR was not specifically identified in those studies, it is plausible that FGR expression could be upregulated as part of the ABC-DLBCL transcriptional program governed by factors like SPIB or NF-κB. Although in our single-center IHC cohort FGR-high frequency did not differ significantly by COO, the survival detriment associated with FGR-high was most pronounced within non-GCB disease. Importantly, external validation in the transcriptomic dataset GSE31312 showed significantly higher FGR mRNA levels in ABC compared with GCB DLBCL and a higher odds of ABC subtype with increasing FGR expression. Further laboratory studies are needed to elucidate the upstream regulators of FGR in DLBCL. It would be interesting to examine tumor gene expression datasets to see if FGR mRNA correlates with ABC-associated gene signatures. Additionally, the relationship between FGR and MYD88 mutation status could be explored, given that HCK is known to be upregulated by the mutant MYD88 pathway in lymphomas (36, 38). If FGR is similarly induced by TLR/MYD88 or BCR signaling, it would strengthen the mechanistic link between FGR expression and ABC-DLBCL’s oncogenic circuitry.

As a SFK, dysregulated FGR signaling could plausibly contribute to therapy resistance when overexpressed or aberrantly activated. Unlike receptor kinases, SFKs lack ligand-binding domains and are regulated by intramolecular interactions (SH2-SH3 domain-mediated autoinhibition) and phosphorylation of a C-terminal tyrosine (26). Overexpression of an SFK can lead to increased collisions and activation, bypassing the normal autoinhibitory mechanisms. FGR activation has been shown to have oncogenic potential in experimental systems. For example, Shu et al. demonstrated that enforcing an active conformation of FGR in hematopoietic cells (by fusing FGR to an oligomerization domain or introducing a “gatekeeper” mutation) caused growth-factor independent proliferation and more aggressive leukemia in mice (28). Notably, those FGR-activated cells exhibited enhanced phosphorylation of downstream survival pathways such as mTORC1/S6K and FAK, which are pathways also implicated in DLBCL cell survival and interaction with the microenvironment (39). It is conceivable that DLBCL tumors with high FGR expression have some degree of autonomous SFK signaling that promotes cell proliferation, survival, and possibly migration. This could explain their poorer response to standard chemotherapy-essentially, FGR-high cells might be more “primed” to activate survival pathways (like NF-κB, STAT3, AKT) in response to stress, including drug treatment. In support of this idea, our data showed that high FGR expression correlated with a higher risk of primary refractory disease. Although we did not directly investigate FGR’s functional effects in this study, our clinical findings align with a model in which FGR overexpression drives more aggressive, therapy-resistant disease.

### 4.3 Clinical applications and future directions

From a clinical perspective, FGR is measurable by routine IHC on FFPE diagnostic tissue and could be incorporated into pathology workflows if validated. In our cohort, FGR showed ROC discrimination at 5 years that was comparable to IPI for both 5-year RR and 5-year death endpoints, suggesting potential complementarity with existing clinical indices. For example, a patient with an otherwise low-intermediate IPI but a tumor that is FGR-high might be considered for more intensive or novel frontline therapy, or closer monitoring post-treatment, given the higher risk of relapse. Conversely, an IPI-high patient whose tumor lacks FGR might have outcomes better than expected and could potentially avoid overtreatment (40). These hypotheses would need prospective evaluation. Notably, with the recent advent of immunotherapy and targeted agents (such as CAR T-cells and bispecific antibodies) for relapsed DLBCL, early identification of high-risk patients who may need alternative first-line strategies is increasingly important (41–43). Biomarkers such as FGR could facilitate personalized, risk-adapted initial treatment approaches, with early integration of kinase inhibitors or immunomodulatory therapies in patients at high risk (44).

External validation strengthens the biological generalizability of the COO association (higher FGR in non-GCB), but the prognostic signal at the mRNA level was not uniformly significant, with only a trend toward worse OS in the highest quartile of expression. This difference highlights important sources of heterogeneity across cohorts and platforms (protein vs mRNA, cutpoint definition, treatment context, and clinical-risk distribution). Future multi-center studies with larger sample sizes and broader real-world risk distributions will be necessary to determine how best to integrate FGR into prognostic frameworks, and whether its clinical utility lies primarily in identifying high-risk disease within non-GCB populations or in combination with other markers.

Our findings also raise the question of whether targeting FGR itself could be therapeutically beneficial. SFKs have long been considered drug targets in cancer, and several multi-kinase inhibitors hit broad members of the SFK family. However, selectively targeting a single SFK has been challenging due to the conserved kinase domains. Interestingly, a novel small-molecule inhibitor TL02–59 was recently reported to selectively inhibit FGR kinase activity with nanomolar potency and showed efficacy in preclinical AML models (45). In an AML xenograft, TL02–59 treatment eradicated FGR-expressing leukemia cells and prolonged survival (27). Even existing drugs like dasatinib, while not specific to FGR, could potentially suppress FGR along with other kinases (46). However, whether FGR-high DLBCL is therapeutically tractable via SFK inhibition remains unknown and warrants dedicated preclinical evaluation, ideally integrating FGR expression as a biomarker for patient selection.

### 4.4 Limitations

This study has several limitations. First, it is a retrospective, single-center analysis with a modest sample size (n = 91) and a limited number of death events, which increases uncertainty in effect estimates and raises the possibility of model instability and overfitting. Second, all patients had ECOG 0-1, the mean age was relatively young, and IPI scores were skewed toward lower risk. These features may limit generalizability to real-world, unselected DLBCL populations and may influence the apparent prognostic impact of FGR. Third, comprehensive genetic subtyping was not available; FISH for MYC/BCL2/BCL6 rearrangements was not routinely performed during the study period, and MYC/BCL2 double-expressor status was not consistently assessed. Therefore, inadvertent inclusion of high-grade B-cell lymphoma with rearrangements or unmeasured double-expressor biology could confound prognostic associations independent of FGR. Fourth, although the IRS ≥ 2 cutpoint was supported by ROC/Youden exploration and selected to be clinically practical, IHC scoring remains semi-quantitative and may be affected by pre-analytic variability and inter-observer interpretation. Finally, mechanistic experiments were not performed; thus, we cannot conclusively prove that FGR overexpression causes treatment resistance or simply co-associates with other causal factors. *In vitro* and *in vivo* studies silencing or overexpressing FGR in DLBCL cells would be valuable to determine its role in cell growth, survival, and drug response.

In conclusion, elevated tumor-cell FGR protein expression is associated with increased treatment failure and inferior outcomes in DLBCL, with a more evident signal in non-GCB disease. These findings support prospective validation in larger, less selected cohorts and motivate mechanistic studies to clarify whether FGR is a driver of aggressive biology and a tractable therapeutic vulnerability.

## Data Availability

The raw data supporting the conclusions of this article will be made available by the authors, without undue reservation.

## References

[1] Wang SS . Epidemiology and etiology of diffuse large B-cell lymphoma. Semin Hematol. (2023) 60:255–66. doi: 10.1053/J.SEMINHEMATOL.2023.11.004. PMID: 38242772 PMC10962251

[2] Jakobsen LH Øvlisen AK Severinsen MT Bæch J Kragholm KH Glimelius I . Patients in complete remission after R-CHOP(-like) therapy for diffuse large B-cell lymphoma have limited excess use of health care services in Denmark. Blood Cancer J. (2022) 12:1–7. doi: 10.1038/S41408-022-00614-8. PMID: 35087026 PMC8795387

[3] He MY Kridel R . Treatment resistance in diffuse large B-cell lymphoma. Leukemia. (2021) 35:2151–65. doi: 10.1038/S41375-021-01285-3. PMID: 34017074

[4] Crump M Neelapu SS Farooq U Van Den Neste E Kuruvilla J Westin J . Outcomes in refractory diffuse large B-cell lymphoma: results from the international SCHOLAR-1 study. Blood. (2017) 130:1800. doi: 10.1182/BLOOD-2017-03-769620. PMID: 28774879 PMC5649550

[5] Wang Y Shi Q Shi ZY Tian S Zhang MC Shen R . Biological signatures of the International Prognostic Index in diffuse large B-cell lymphoma. Blood Adv. (2024) 8:1587–99. doi: 10.1182/bloodadvances.2023011425. PMID: 38170757 PMC10987882

[6] Ruppert AS Dixon JG Salles G Wall A Cunningham D Poeschel V . International prognostic indices in diffuse large B-cell lymphoma: a comparison of IPI, R-IPI, and NCCN-IPI. Blood. (2020) 135:2041–8. doi: 10.1182/BLOOD.2019002729. PMID: 32232482

[7] Jelicic J Juul-Jensen K Bukumiric Z Roost Clausen M Ludvigsen Al-Mashhadi A Pedersen RS . Prognostic indices in diffuse large B-cell lymphoma: a population-based comparison and validation study of multiple models. Blood Cancer J. (2023) 13:157. doi: 10.1038/S41408-023-00930-7. PMID: 37833260 PMC10575851

[8] Liang XJ Song XY Wu JL Liu D Lin BY Zhou HS . Advances in multi-omics study of prognostic biomarkers of diffuse large B-cell lymphoma. Int J Biol Sci. (2022) 18:1313–27. doi: 10.7150/IJBS.67892. PMID: 35280688 PMC8898353

[9] Wright GW Huang DW Phelan JD Coulibaly ZA Roulland S Young RM . A probabilistic classification tool for genetic subtypes of diffuse large B cell lymphoma with therapeutic implications. Cancer Cell. (2020) 37:551–568.e14. doi: 10.1016/j.ccell.2020.03.015. PMID: 32289277 PMC8459709

[10] He J Chen Z Xue Q Sun P Wang Y Zhu C . Identification of molecular subtypes and a novel prognostic model of diffuse large B-cell lymphoma based on a metabolism-associated gene signature. J Transl Med. (2022) 20:1–21. doi: 10.1186/S12967-022-03393-9. PMID: 35468826 PMC9036805

[11] Pan M Yang P Wang F Luo X Li B Ding Y . Whole transcriptome data analysis reveals prognostic signature genes for overall survival prediction in diffuse large B cell lymphoma. Front Genet. (2021) 12:648800. doi: 10.3389/FGENE.2021.648800. PMID: 34178023 PMC8220154

[12] Zhang Z Zhao C Yang S Lu W Shi J . A novel lipid metabolism-based risk model associated with immunosuppressive mechanisms in diffuse large B-cell lymphoma. Lipids Health Dis. (2024) 23(1):20. doi: 10.1186/S12944-024-02017-Z. PMID: 38254162 PMC10801940

[13] Cui Y Leng C . A glycolysis-related gene signatures in diffuse large B-Cell lymphoma predicts prognosis and tumor immune microenvironment. Front Cell Dev Biol. (2023) 11:1070777. doi: 10.3389/FCELL.2023.1070777. PMID: 36755971 PMC9899826

[14] Liang X Hu R Li Q Wang C Liu Y . Prognostic factors for diffuse large B-cell lymphoma: clinical and biological factors in the rituximab era. Exp Hematol. (2023) 122:1–9. doi: 10.1016/j.exphem.2023.03.003. PMID: 36933759

[15] Guan X Wang Y Fang T Wang J Li R Hao M . Lymphoma cell-driven IL-16 is expressed in activated B-cell-like diffuse large B-cell lymphomas and regulates the pro-tumor microenvironment. Haematologica. (2025) 110:425–38. doi: 10.3324/HAEMATOL.2024.285304. PMID: 39323416 PMC11788625

[16] Alduaij W Collinge B Ben-Neriah S Jiang A Hilton LK Boyle M . Molecular determinants of clinical outcomes in a real-world diffuse large B-cell lymphoma population. Blood. (2023) 141:2493–507. doi: 10.1182/BLOOD.2022018248. PMID: 36302166

[17] Sartori G Napoli S Cascione L Chung EYL Priebe V Arribas AJ . ASB2 is a direct target of FLI1 that sustains NF-κB pathway activation in germinal center-derived diffuse large B-cell lymphoma. J Exp Clin Cancer Res. (2021) 40:357. doi: 10.1186/S13046-021-02159-3. PMID: 34763718 PMC8582153

[18] Hans CP Weisenburger DD Greiner TC Gascoyne RD Delabie J Ott G . Confirmation of the molecular classification of diffuse large B-cell lymphoma by immunohistochemistry using a tissue microarray. Blood. (2004) 103:275–82. doi: 10.1182/BLOOD-2003-05-1545. PMID: 14504078

[19] Chapuy B Stewart C Dunford AJ Kim J Kamburov A Redd RA . Molecular subtypes of diffuse large B cell lymphoma are associated with distinct pathogenic mechanisms and outcomes. Nat Med. (2018) 24:679–90. doi: 10.1038/S41591-018-0016-8. PMID: 29713087 PMC6613387

[20] Schmitz R Wright GW Huang DW Johnson CA Phelan JD Wang JQ . Genetics and pathogenesis of diffuse large B-cell lymphoma. N Engl J Med. (2018) 378:1396–407. doi: 10.1056/NEJMOA1801445. PMID: 29641966 PMC6010183

[21] Akkus C Demircioglu S Erten R Dagli SC Dogan A . Evaluation of PD-1 / PD-L1 expressions in patients with diffuse large B cell lymphoma and chronic lymphocytic leukemia. Selcuk Med J. (2024) 40(2):82–87. doi: 10.30733/std.2024.01714

[22] Boggon TJ Eck MJ . Structure and regulation of Src family kinases. Oncogene. (2004) 23:7918–27. doi: 10.1038/SJ.ONC.1208081. PMID: 15489910

[23] Lantermans HC Minderman M Kuil A Kersten MJ Pals ST Spaargaren M . Identification of the SRC-family tyrosine kinase HCK as a therapeutic target in mantle cell lymphoma. Leukemia. (2021) 35:881–6. doi: 10.1038/S41375-020-0934-6. PMID: 32591642 PMC7932922

[24] Tesch H Abts H Jucker M Mays K Lenoir G Diehl V . Expression of c-fgr in EBV positive and negative B cell tumors. Leukemia. (1989) 3:897–8. 2555634

[25] Cheah MSC Ley TJ Tronick SR Robbins KC . fgr proto-oncogene mRNA induced in B lymphocytes by Epstein-Barr virus infection. Nature. (1986) 319:238–40. doi: 10.1038/319238a0. PMID: 3003578

[26] Shen K Moroco JA Patel RK Shi H Engen JR Dorman HR . The Src family kinase Fgr is a transforming oncoprotein that functions independently of SH3-SH2 domain regulation. Sci Signal. (2018) 11(553):eaat5916. doi: 10.1126/SCISIGNAL.AAT5916. PMID: 30352950

[27] Du S Alvarado JJ Wales TE Moroco JA Engen JR Smithgall TE . ATP-site inhibitors induce unique conformations of the acute myeloid leukemia-associated Src-family kinase, Fgr. Structure. (2022) 30:1508–1517.e3. doi: 10.1016/J.STR.2022.08.008. PMID: 36115344 PMC9637690

[28] Shu ST Chen L Gonzalez-Areizaga G Smithgall TE . Constitutive activation of the Src-family kinases Fgr and Hck enhances the tumor burden of acute myeloid leukemia cells in immunocompromised mice. Sci Rep. (2025) 15:174. doi: 10.1038/S41598-024-83740-6. PMID: 39747387 PMC11697302

[29] Joosten M Ginzel S Blex C Schmidt D Gombert M Chen C . A novel approach to detect resistance mechanisms reveals FGR as a factor mediating HDAC inhibitor SAHA resistance in B-cell lymphoma. Mol Oncol. (2016) 10:1232–44. doi: 10.1016/j.molonc.2016.06.001. PMID: 27324824 PMC5423193

[30] Patel M Leevers SJ Brickell PM . Regulation of C‐FGR proto‐oncogene expression in epstein‐barr virus infected B‐cell lines. Int J Cancer. (1990) 45:342–6. doi: 10.1002/ijc.2910450222. PMID: 2154410

[31] Beck T Weikel W Brumm C Wilkens C Pollow K Knapstein P-G . Immunohistochemical detection of hormone receptors in breast carcinomas (ER-ICA, PgR-ICA): prognostic usefulness and comparison with the biochemical radioactive-ligand-binding assay (DCC). Gynecol Oncol. (1994) 53:220–7. doi: 10.1006/GYNO.1994.1119. PMID: 8188083

[32] DeLong ER DeLong DM Clarke-Pearson DL . Comparing the areas under two or more correlated receiver operating characteristic curves: a nonparametric approach. Biometrics. (1988) 44:837. doi: 10.2307/2531595 3203132

[33] Hwang J Suh CH Kim KW Kim HS Kim AI Craig JW . The incidence and treatment response of double expression of myc and bcl2 in patients with diffuse large b-cell lymphoma: a systematic review and meta-analysis. Cancers (Basel). (2021) 13(13):3369. doi: 10.3390/CANCERS13133369. PMID: 34282799 PMC8268769

[34] Ma Z Niu J Cao Y Pang X Cui W Zhang W . Clinical significance of ‘double-hit’ and ‘double-expression’ lymphomas. J Clin Pathol. (2020) 73:126–38. doi: 10.1136/JCLINPATH-2019-206199. PMID: 31615842

[35] Zhuo Y Zhang D . Recent advancements in double-expressor lymphoma: novel therapeutic approaches and prospects. Oncologist. (2025) 30:oyaf085. doi: 10.1093/ONCOLO/OYAF085. PMID: 40525911 PMC12200234

[36] Munshi M Liu X Kofides A Tsakmaklis N Guerrera ML Hunter ZR . A new role for the SRC family kinase HCK as a driver of SYK activation in MYD88 mutated lymphomas. Blood Adv. (2022) 6:3332–8. doi: 10.1182/BLOODADVANCES.2021006147. PMID: 35255496 PMC9198919

[37] Flümann R Hansen J Pelzer BW Nieper P Lohmann T Kisis I . Distinct genetically determined origins of Myd88/BCL2-driven aggressive lymphoma rationalize targeted therapeutic intervention strategies. Blood Cancer Discov. (2023) 4:78–97. doi: 10.1158/2643-3230.BCD-22-0007. PMID: 36346827 PMC9816818

[38] Yang G Buhrlage SJ Tan L Liu X Chen J Xu L . HCK is a survival determinant transactivated by mutated MYD88, and a direct target of ibrutinib. Blood. (2016) 127:3237–52. doi: 10.1182/BLOOD-2016-01-695098. PMID: 27143257

[39] Zou Z Tao T Li H Zhu X . MTOR signaling pathway and mTOR inhibitors in cancer: progress and challenges. Cell Biosci. (2020) 10:1–11. doi: 10.1186/s13578-020-00396-1, PMID: 32175074 PMC7063815

[40] Chen W Liang W He Y Liu C Chen H Lv P . Immune microenvironment-related gene mapping predicts immunochemotherapy response and prognosis in diffuse large B-cell lymphoma. Med Oncol. (2022) 39:44. doi: 10.1007/S12032-021-01642-3. PMID: 35092504

[41] Locke FL Miklos DB Jacobson CA Perales M-A Kersten M-J Oluwole OO . Axicabtagene ciloleucel as second-line therapy for large B-cell lymphoma. N Engl J Med. (2022) 386:640–54. doi: 10.1056/NEJMOA2116133. PMID: 34891224

[42] Dickinson MJ Carlo-Stella C Morschhauser F Bachy E Corradini P Iacoboni G . Glofitamab for relapsed or refractory diffuse large B-cell lymphoma. N Engl J Med. (2022) 387:2220–31. doi: 10.1056/NEJMOA2206913. PMID: 36507690

[43] Ibrahiam AT Geddada S Ullah N Al-Qassab ZM Ahmed O Khan S . Chimeric antigen receptor (CAR) T-cell therapy in the treatment of diffuse large B-cell lymphoma (DLBCL): a systematic review. Cureus. (2024) 16(12):e75854. doi: 10.7759/CUREUS.75854. PMID: 39822464 PMC11738109

[44] Tilly H Morschhauser F Sehn LH Friedberg JW Trněný M Sharman JP . Polatuzumab vedotin in previously untreated diffuse large B-cell lymphoma. N Engl J Med. (2022) 386:351–63. doi: 10.1056/NEJMOA2115304. PMID: 34904799 PMC11702892

[45] Weir MC Shu ST Patel RK Hellwig S Chen L Tan L . Selective inhibition of the myeloid Src-family kinase Fgr potently suppresses AML cell growth *in vitro* and *in vivo*. ACS Chem Biol. (2018) 13:1551. doi: 10.1021/ACSCHEMBIO.8B00154. PMID: 29763550 PMC6130198

[46] Hmedat ANA Doondeea J Ebner D Feller SM Lewitzky M . The Src family kinase inhibitor drug Dasatinib and glucocorticoids display synergistic activity against tongue squamous cell carcinoma and reduce MET kinase activity. Cell Commun Signaling. (2025) 23:293. doi: 10.1186/S12964-025-02129-8. PMID: 40537792 PMC12180234

