# Supplementary material for: Elevated FGR protein expression identifies a high-risk subset of diffuse large B-cell lymphoma and a potential therapeutic target
Source: Front Oncol. 2026 Apr 16;16:1688949. doi: 10.3389/fonc.2026.1688949 (PMC13128391; doi:10.3389/fonc.2026.1688949)
Supplement: Supplementary file 1 [file DataSheet1.docx]

Supplementary Material

# Supplementary Data

Supplementary Material should be uploaded separately on submission. Please include any supplementary data, figures and/or tables.

Supplementary material is not typeset so please ensure that all information is clearly presented, the appropriate caption is included in the file and not in the manuscript, and that the style conforms to the rest of the article.

# Supplementary Figures and Tables

For more information on Supplementary Material and for details on the different file types accepted, please see [here](https://www.frontiersin.org/guidelines/author-guidelines#supplementary-material).

## Supplementary Tables

| PFS | | | | | | OS | | | | | |
| --- | --- | --- | --- | --- | --- | --- | --- | --- | --- | --- | --- |
| IRS | Sensitivity | Specificity | PPV | NPV | Youden | IRS | Sensitivity | Specificity | PPV | NPV | Youden |
| 1 | 1.000 | 0.000 | 0.231 |  | 0.000 | 1 | 1.000 | 0.000 | 0.143 |  | 0.000 |
| 2 | 0.667 | 0.500 | 0.286 | 0.833 | 0.167 | 2 | 0.692 | 0.487 | 0.184 | 0.905 | 0.179 |
| 3 | 0.429 | 0.657 | 0.273 | 0.793 | 0.086 | 3 | 0.385 | 0.641 | 0.152 | 0.862 | 0.026 |
| 4 | 0.143 | 0.757 | 0.150 | 0.746 | -0.100 | 4 | 0.077 | 0.756 | 0.050 | 0.831 | -0.167 |
| 4.5 | 0.143 | 0.771 | 0.158 | 0.750 | -0.086 | 4.5 | 0.077 | 0.769 | 0.053 | 0.833 | -0.154 |
| 6 | 0.095 | 0.871 | 0.182 | 0.763 | -0.033 | 6 | 0.077 | 0.872 | 0.091 | 0.850 | -0.051 |
| 8 | 0.048 | 0.943 | 0.200 | 0.767 | -0.010 | 8 | 0.077 | 0.949 | 0.200 | 0.860 | 0.026 |

**Supplementary Table S1.** Youden-index analyses for determining the optimal IRS threshold to predict 5-year PFS and OS.

## Supplementary Figures

**(b)**

**(a)**

**Supplementary Figure S1.** Survival analyses of GSE31312 stratified by FGR mRNA expression.
